# Co-occurrence of Moyamoya syndrome and Kartagener syndrome caused by the mutation of *DNAH5* and *DNAH11*: a case report

**DOI:** 10.1186/s12883-020-01895-x

**Published:** 2020-08-26

**Authors:** Lili Zhang, Xungang Feng, Junhu Zhang, Yanlei Hao, Yuzhong Wang

**Affiliations:** 1 Department of Neurology, Affiliated Hospital of Jining Medical University, 89 Guhuai Road, Jining City, 272029 Shandong Province China; 2 Medical Research Centre, Affiliated Hospital of Jining Medical University, 89 Guhuai Road, Jining City, 272029 Shandong Province China

**Keywords:** Kartagener syndrome, Primary ciliary dyskinesia, Moyamoya syndrome, Dynein axonemal heavy chain 5, Dynein axonemal heavy chain 11

## Abstract

**Background:**

Kartagener syndrome is an autosomal recessive inherited disorder of primary ciliary dyskinesia. Moyamoya syndrome refers to a moyamoya angiopathy associated with other neurological and/or extra-neurological symptoms, or due to a well identified acquired or inherited cause. We herein reported a case of a 48-year-old woman who was favored the diagnosis of Kartagener syndrome and moyamoya syndrome. The whole genome sequencing and bioinformatics analysis showed a homozygotic nonsense mutation in the dynein, axonemal, heavy chain (*DNAH*) 5 gene, and heterozygotic missense mutation in the *DNAH11* gene. This is the first report of the co-occurrence of the two rare diseases.

**Case presentation:**

A case of a 48-year-old woman was presented with hemiplegia and slurred speech. The magnetic resonance imaging of the brain confirmed acute cerebral infarction in the right basal ganglia region, semi-oval center, insular lobe, and frontal parietal lobe. The electrocardiogram showed inverted “P” waves in L1 and AVL on left-sided chest leads and computed tomography scan of the chest showed bronchiectasis changes, cardiac shadow and apex on the right side, and situs inversus of aortic arch position. The digital subtraction angiography showed inversion of the aortic arch, and bilateral internal carotid arteries are occluded from the ophthalmic segment. The clinical, radiological, and laboratory findings made the diagnosis of Kartagener syndrome and moyamoya syndrome. The whole genome sequencing and bioinformatics analysis showed a homozygotic nonsense mutation in *DNAH5* gene, and heterozygotic missense mutation in the *DNAH11* gene.

**Conclusion:**

The combined mutation of *DNAH5* and *DNAH11* may lead to the overlapping dysfunction of motile and nonmotile cilia, which contribute to the co-occurrence of Kartagener syndrome and moyamoya syndrome. Our report deserves further confirm by more case reports.

## Background

Kartagener syndrome is a rare hereditary disease characterized by the clinical triad of bronchiectasis, situs inversus and chronic sinusitis, and is a subset of primary ciliary dyskinesia (PCD) [1]. The estimated prevalence of Kartagener syndrome is at 1/32,000 births [2]. Moyamoya angiopathy is a chronic cerebrovascular occlusive disease characterized by progressive stenoses or occlusion of the bilateral terminal portions of the internal carotid and the proximal anterior and middle cerebral arteries, followed by the formation of compensatory collateral vessels at the base of the brain [3]. Moyamoya disease is an isolated and primary moyamoya angiopathy; however, moyamoya syndrome (MMS) occurs in association with some acquired conditions, such as cranial therapeutic radiation, or genetic disorders [4, 5]. Worldwide, the incidence of moyamoya disease and syndrome showed significant geographic difference. From 2000 to 2007, an epidemiology study of moyamoya disease conducted in Nanjing, China reported a prevalence of 3.92 per 100,000 [6]. Although MMS complicated by other genetic disorders was frequently reported [5], co-occurrence of Kartagener syndrome and MMS has not been reported. We herein firstly reported a case of co-occurrence of Kartagener syndrome and MMS, who had a homozygotic nonsense mutation in *DNAH5* gene, and heterozygotic missense mutation in the *DNAH11* gene.

## Case presentation

A 48-year-old stout woman farmer presented to our hospital because of 1 day history of weakness of left limb and barylalia on May 20, 2018. She had a history of rhinosinusitis, chronic bronchitis, and hypertension. She had no history of cardiovascular and cerebrovascular events and had no use of tobacco and alcohol. Her parents were not close relatives and she had a healthy daughter. Her mother died suddenly many years ago without definite cause. Her father and siblings are healthy and live far away in Yunnan province, the Southwest China. Her vital signs at entry included respiratory rate at 19 per min, heart rate at 79 per min, blood pressure at 130/79 mmHg, and body temperature at 36.7 °C. The physical examination showed motor aphasia, left central facial and lingual palsy as well as the left limb weakness. The National Institute of Health stroke scale was four. There were dry lung rales on both sides. Heart sounds were more pronounced at the right sternal border. The computed tomography scan of brain at emergency excluded the hemorrhage. A probable diagnosis of cerebral infarction was made.

**Fig. 1:**
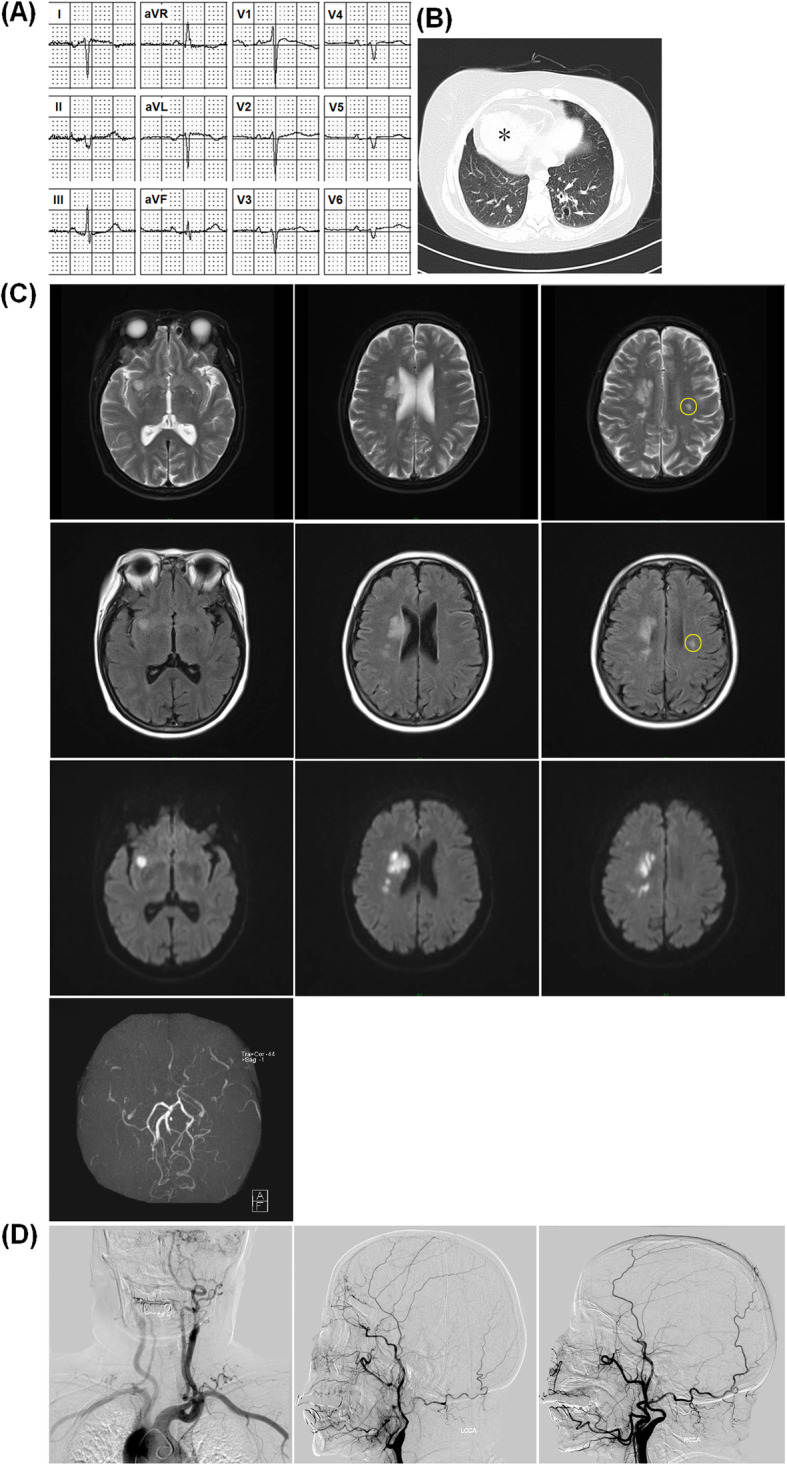
Electrocardiogram and imaging findings in the case with co-occurrence of moyamoya syndrome and Kartagener syndrome. **a**, Electrocardiogram showed inverted “P” waves in L1 and AVL on left-sided chest leads. **b**, Computed tomography scan of the chest revealed bronchiectasis changes, and the cardiac shadow and apex on the right side, and aortic arch, situs inversus. **c**, The brain magnetic resonance imaging showed acute cerebral infarction in the right basal ganglia region, semi-oval center, insular lobe and frontal parietal lobe. The left coronal areas and left semi-oval central area has old ischemic area (circle). Magnetic resonance angiography revealed severe stenosis at the bilateral terminal portion of the internal carotid arteries while the bilateral middle cerebral arteries and anterior cerebral arteries had almost disappeared. **d**, Digital subtraction angiography showed inversion of the aortic arch, bilateral internal carotid arteries are occluded at the ophthalmic segment

Her electrocardiogram showed inverted “P” waves in L1 and AVL on left-sided chest leads (Fig. 1a). Computed tomography scan of the chest revealed bronchiectasis changes, and the cardiac shadow and apex on the right side, and aortic arch, situs inversus (Fig. 1b). The cardiac ultrasound showed heart inversus. Further laboratory test showed normal blood routine test, C reactive protein, erythrocyte sedimentation rate and seronegative for anti-nuclear antibody, anti-neutrophil cytoplasmic antibody, anti-CCP antibody, rheumatoid factor, anti-HIV and anti-Syphilis antibodies. The levels of cholesterol, low-density lipoprotein, and high-density lipoprotein are normal, 4.58 mmol/L, 2.67 mmol/L and 1.13 mmol/L, respectively. The magnetic resonance imaging of brain showed acute cerebral infarction in the right basal ganglia region, semi-oval center, insular lobe, frontal parietal lobe and sinusitis as well as old ischemic area in the left coronal areas and left semi-oval central area (Fig. 1c). In addition, magnetic resonance angiography of brain revealed severe stenosis at the bilateral terminal portion of the internal carotid arteries, while the bilateral middle cerebral arteries and anterior cerebral arteries almost disappeared. A further digital subtraction angiography (Fig. 1d) showed inversion of the aortic arch, and bilateral internal carotid arteries are occluded at the ophthalmic segment.

**Fig. 2:**
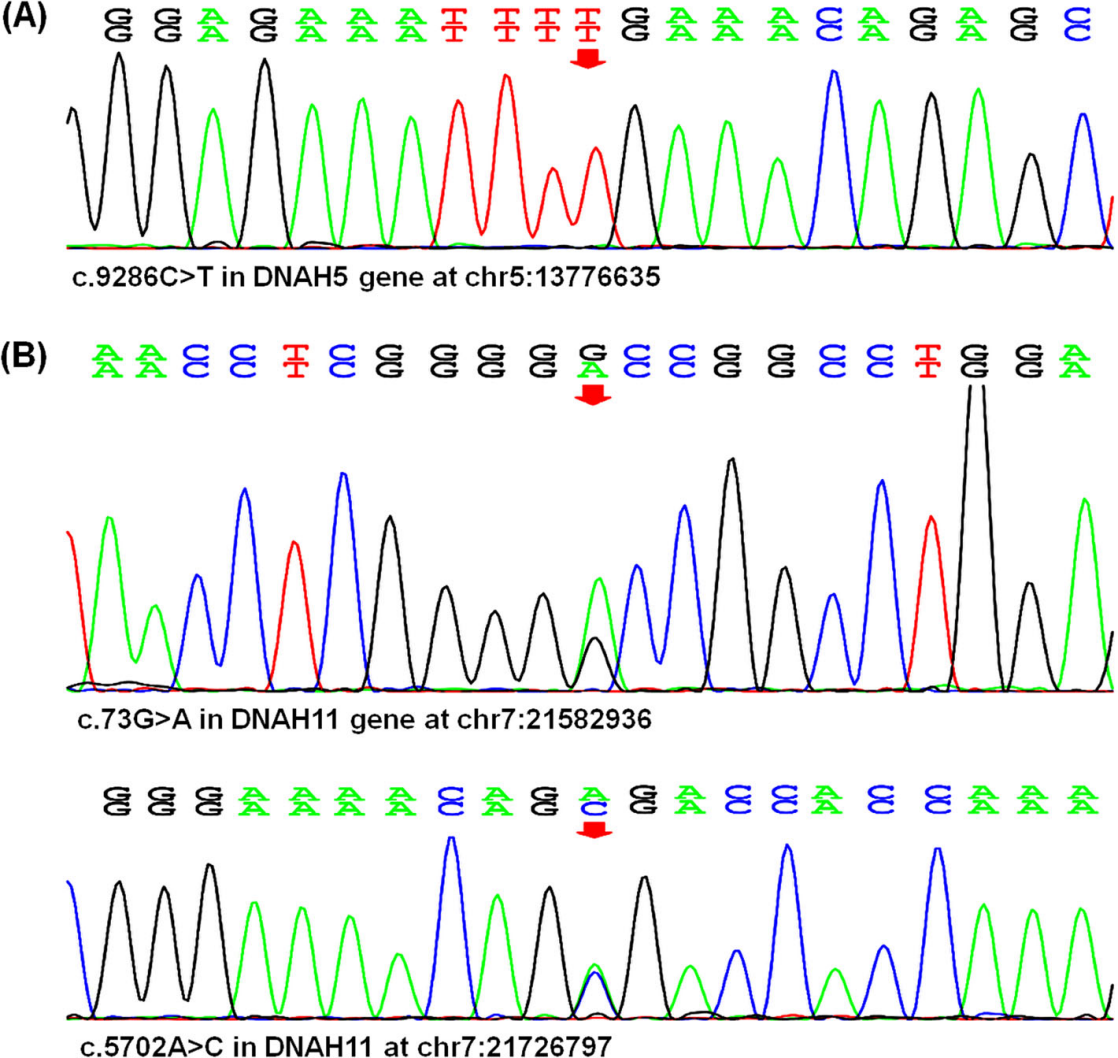
Co-occurrence of moyamoya syndrome and Kartagener syndrome associated with mutations in *DNAH5* and *DNAH11* gene. The whole genome sequencing analysis showed a nonsense mutation c.9286C > T (p.Arg3096Ter) in the *DNAH5* gene (**a**) and heterozygotic missense mutation including c.73G > A (p.Ala25Thr) and c.5702A > C (p.Glu1901Ala) in the *DNAH11* gene (**b**)

The whole genome sequencing and bioinformatics analysis showed a homozygotic nonsense mutation c.9286C > T (p.Arg3096Ter) at Exon 55 in the dynein, axonemal, heavy chain (*DNAH*) 5 gene (Fig. 2a), and heterozygotic missense mutation c.73G > A (p.Ala25Thr) at Exon 1, c.5702A > C (p.Glu1901Ala) at Exon 33 in the *DNAH11* gene (Fig. 2b). Finally, a diagnosis of cerebral infarction, MMS and Kartagener syndrome was made.

She was treated with aspirin, rosuvastatin, intravenous injection of vinpocetine and other supportive treatment. She rejected further evaluation of brain perfusion because of financial reasons. Ten days later, her symptoms improved with the National Institute of Health stroke scale of two and then she was discharged. A follow-up at 18 months after onset showed no sequelae of cerebral infarction, modified Rankin Scale was 0.

## Discussion and conclusions

Our case is a confirmed co-occurrence of moyamoya syndrome and Kartagener syndrome. Kartagener syndrome occurs in association with many genes encoding the proteins that are important to the structure and function of cilia. There are two types of cilia, motile and nonmotile cilia, the former beats rhythmically and function in cellular and organismal locomotion to drive fluid transport over epithelia while the later type serves as sensory organelles [7]. Motile ciliopathies, such as PCD, causing failure of mucus clearance and chronic airway diseases, are associated with defects of laterality, fertility and brain development [8]. Among 34 cilia mutations causing laterality defects, 22 genes modulate the nonmotile cilia (*CC2D2A*, *ANKS6*, *NEK8*, *MKS1*, *CEP290*, *BICC1*) while the mutations of other 12 genes will result in the disorder of the motile cilia (*DNAH5*, *DNAH11*, *DNAI1*, *DAW1*, *ARMC4*, *CCDC151*, *DRC1*, *CCDC39*, *DYXC1X1*, *DNAAF3*) [9]. For this case, the whole genome sequencing showed a nonsense mutation c.9286C > T in the *DNAH5* gene, which results in early termination of protein translation. The heterozygotic missense mutation c.73G > A (p.Ala25Thr) at Exon 1, c.5702A > C (p.Glu1901Ala) at Exon 33 in the *DNAH11* gene may result in the protein dysfunction.

The MMS-related genetic disorders have been well summarized by Luisa et al. [5]. The mutations in *DNAH5* and *DNAH11* gene accounts well for the development of PCD in this case; however, the roles of the two gene mutations in mechanism of MMS remains elusive. Although nonmotile and motile ciliopathies are respectively associated with some definite mutations in cilia protein-encoding genes, recent studies indicated that the boundary between motile and nonmotile cilia-related ciliopathies and symptoms may be not clear [10]. For example, patients with mutation of *CEP290*, which is considered to only affect the nonmotile cilia, had abnormal respiratory cilia and respiratory symptoms [11]. Previous research suggested mutations of *DNAH5* and *DNAH11* result in the PCD and respiratory symptoms; however, recent evidences demonstrated significant association between mutations of *DNAH5* and *DNAH11* and the cardiovascular diseases, which are usually caused by defects in nonmotile cilia [12, 13]. A recent report confirms that removing nonmotile cilia from the vascular branch points causes abnormal fluid-flow responses that contribute to the atherosclerosis [14]. Additionally, the most common gene mutation in *DNAH11* is heterozygotic missense mutation [13], which has strong association with low density lipoprotein levels and ischemic stroke [15, 16]. However, our case has normal levels of blood lipids, which does not support the *DNAH11* gene mutation-related blood lipid abnormality as the cause of MMS in our case. It is possible that, for our case, mutations in *DNAH5* and *DNAH11* gene may cause the dysfunction of both nonmotile and motile cilia, which may contribute to abnormal fluid-flow responses and atherosclerosis, resulting in MMS. A surgical intervention should be considered for patients with symptomatic MMS. However, our case rejected further evaluation of brain perfusion and surgical treatment because of financial reasons.

To sum up, this is the first report of co-occurrence of MMS and Kartagener syndrome, which may be cause by a nonsense mutation in *DNAH5* gene and heterozygotic missense mutation in *DNAH11* gene. Our report deserves further confirmation by more case reports. Further study using gene-edited animal model is necessary to demonstrate the association between MMS and mutations in *DNAH5* and *DNAH11* gene to elucidate the mechanism of co-occurrence of the two diseases.

## Data Availability

Data sharing is not applicable to this article as no datasets were generated or analyzed during the current study.

## Abbreviations

*DNAH*: Dynein, axonemal, heavy chain
PCD: Primary ciliary dyskinesia
MMS: Moyamoya syndrome
